# Radiation dose and signal-to-noise ratio in pediatric head CT: a phantom study comparing photon-counting and energy-integrating detectors

**DOI:** 10.1007/s00234-026-04075-9

**Published:** 2026-06-20

**Authors:** Laura Valentina Klüner, Sebastian Zensen, Hannah Peuster, Raya Ocker-Serger, Marcel Drews, Denise Bos, Cornelius Deuschl, Lale Umutlu, Michael Forsting, Johannes Haubold, Marcel Opitz

**Affiliations:** 1 Institute of Diagnostic and Interventional Radiology and Neuroradiology, Essen University Hospital, Essen, Germany; 2 Faculty of Medicine, University of Duisburg-Essen, Essen, Germany; 3 Institute of Diagnostic and Interventional Radiology, University Hospital of Zurich, Zurich, Switzerland

**Keywords:** Photon-counting CT, Pediatric, Head, Image quality, EID-CT, SNR

## Abstract

**Purpose:**

This study evaluated the relationship between image quality level (IQL), radiation dose, image noise, and signal-to-noise ratio (SNR) in pediatric head CT, comparing energy-integrating detector CT (EID-CT) and photon-counting CT (PCCT) across age-specific phantoms.

**Methods:**

Head CT scans of 1-, 5-, and 10-year-old anthropomorphic phantoms were performed across a range of IQLs on EID-CT and PCCT. For each IQL, radiation dose, image noise, and SNR were recorded and compared between the two systems.

**Results:**

PCCT required lower CTDI_vol_ across all phantoms (*p* < 0.05). In the clinically relevant IQL-range (IQL 200–300) the 10-year-old phantom showed the greatest relative radiation dose reduction using PCCT (9%), compared to 0.45% in the 1-year-old phantom. Organ doses were highest in the 1-year-old phantom for red bone marrow and skin with no significant differences between the two systems. PCCT offered significantly higher SNR than EID-CT for brain parenchyma (*p* < 0.03) and bone (*p* = 0.012). Image noise was significantly lower in PCCT for brain parenchyma in the 1- and 5-year-old phantoms (*p* < 0.03) and for bone in the 1-year-old phantom (*p* = 0.012). Over the full IQL-range (1-300) the average SNR was up to ~ 20% higher for brain parenchyma and ~ 50% higher for bone with PCCT than with EID-CT for all phantoms.

**Conclusion:**

PCCT offers superior SNR for brain and bone while reducing radiation dose, with the largest radiation dose benefit in older pediatric phantoms, supporting optimized pediatric neuroimaging.

**Supplementary Information:**

The online version contains supplementary material available at 10.1007/s00234-026-04075-9.

## Introduction

The number of CT examinations worldwide has increased and currently accounts for approximately 50% of the global population’s exposure to medical radiation [1]. This is of particular concern in pediatric imaging, where radiation exposure is especially critical. According to the linear no-threshold model, even a single CT scan may increase the lifetime risk of hematological malignancies in children [2]. As a result, radiation protection strategies such as the ‘As Low As Reasonably Achievable’ (ALARA) principle are emphasized to minimize unnecessary radiation exposure [3]. To better monitor and manage radiation exposure, Diagnostic Reference Levels (DRLs) have been established, which are defined as radiation dose levels in medical imaging for typical examinations on standard-sized patients [4]. In recent years, CT technology has evolved substantially. While energy-integrating detectors (EID-CT) use a two-step process to convert X-ray photons into electrical signals, photon-counting CT (PCCT) performs this conversion in a single step. This offers several advantages, including higher spatial resolution due to the absence of inter-detector septa, inherent spectral imaging capabilities, and improved contrast for materials with high atomic numbers [5].

Several studies have analyzed differences between PCCT and EID-CT in terms of radiation dose and image quality. Multiple studies consistently report a significant reduction in image noise with PCCT, both quantitatively and visually [6, 7]. In the field of neuroimaging, comparative studies have examined PCCT versus EID-CT for applications such as skull base visualization, contrast-enhanced imaging, and non-enhanced brain imaging [8]. For high-resolution bone imaging of the skull, PCCT has demonstrated improved spatial resolution and a 20–30% radiation dose reduction in temporal bone imaging compared to EID-CT [9–11]. Similar benefits have been reported for imaging of the paranasal sinuses, where lower radiation doses and higher image quality were achieved [12]. Furthermore, the cribriform plate exhibited higher SNR values on PCCT compared to EID-CT [13]. The improved SNR and lower noise levels with PCCT in bone imaging have also been shown for other skeletal regions, such as cadaveric forearm imaging [14], and for lumbar spine CT, where a significantly lower radiation dose was reported without compromising image quality [15]. In contrast-enhanced neuroimaging, PCCT has shown higher spatial resolution in the depiction of neurovascular structures and greater diagnostic confidence for arterial findings [16, 17]. Additionally, PCCT myelography demonstrated increased sensitivity for the detection of cerebrospinal fluid (CSF)-venous fistulas [18]. For non-contrast neuroimaging, PCCT has outperformed EID-CT in the evaluation of focal brain lesions [19] and in the contrast differentiation between gray and white matter [20].

To enable standardized, system-independent image quality settings prior to scanning, PCCT systems allow the definition of an image quality level (IQL). This parameter replaces the former ‘reference mAs’ and reflects the effective mAs required for an average-sized patient. Studies have demonstrated a linear relationship between IQLs and mAs or dose metrics (e.g., CTDI_vol_, DLP) [21], while the relationship between IQL and signal-to-noise ratio (SNR) appears non-linear [22]. Therefore, clinicians should be aware that increasing radiation dose does not necessarily result in a proportional improvement in SNR. This study aims to evaluate the relationship between IQL, radiation dose, image noise, and SNR in pediatric head CT using standard clinical head imaging protocols on both EID-CT and PCCT systems.

## Methods

The exclusive use of anthropomorphic phantoms exempted this study from ethical approval by the institutional review board.

### Image acquisition and CT protocols

Three different anthropomorphic phantoms (ATOM^®^ Dosimetry Phantoms, CIRS, Norfolk, USA) were used in this study for head CT scans: 10-year-old (height: 140 cm), 5-year-old (height: 110 cm), and 1-year-old (height: 75 cm). These phantoms are anatomically detailed with tissue-equivalent materials that simulate human anatomy and radiological properties [23]. Two CT systems were used for image acquisition: an energy-integrating detector CT (SOMATOM X.ceed, Siemens Healthineers, Germany) and a photon-counting CT (NAEOTOM Alpha, Siemens Healthineers, Germany). IQLs were manually selected for all acquisitions on both systems. They define the targeted image quality by maintaining a consistent CNR at predefined keV levels and ensure harmonized and comparable image quality across different Siemens CT systems. On the PCCT, IQL regulation is performed through the automatic exposure control CARE keV, whereas on the EID-CT it is performed through CARE kV (Siemens Healthineers, Erlangen, Germany). Pediatric phantoms were scanned on both systems. PCCT measurements of the pediatric phantoms have previously been analyzed in the context of age-related differences between adult and pediatric head phantoms on PCCT [24]. The present study investigates a distinct research question by directly comparing these pediatric PCCT measurements with newly acquired matched EID-CT acquisitions. Scan parameters are summarized in Table 1.

**Table 1:** Characteristics of technical scan parameters using standard clinical protocols for EID-CT and PCCT for pediatric patients

Scans on PCCT							
Phantoms	kVp	Reconstructed slice thickness [mm]	Reconstruction kernel	Iterative reconstruction strength	Pitch	Rotation time [s]	Collimation
1-year-old	70	1.5	Brain parenchyma: Hr40 Bone: Hr60	2	0.35	0.5	57.6 mm (144 × 0.4 mm)
5-year-old	70	1.5					
10-year-old	70	1.5					
Scans on EID-CT							
Phantoms	kVp	Reconstructed slice thickness [mm]	Reconstruction kernel	Iterative reconstruction strength	Pitch	Rotation time [s]	Collimation
1-year-old	100	1	Brain parenchyma: Hr40 Bone: Hr60	2	0.55	1	38.4 mm (64 × 0.6 mm)
5-year-old	100						
10-year-old	100						

### Quantitative data and dose assessment

**Fig. 1:**
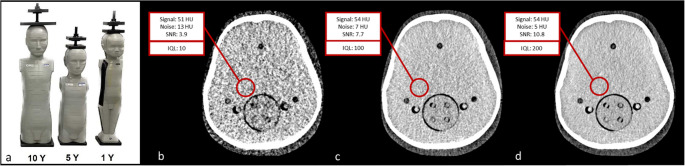
Visualization of the 5 anthropomorphic phantoms and the ROI measurements of brain parenchyma. **a**: Three pediatric phantoms (adult male, adult female, 10 years, 5 years, and 1 year). **b-d**: ROI measurements of brain parenchyma tissue equivalent for IQL 10 (**b**), IQL 100 (**c**) and IQL 200 (**d**) of the 1-year-old phantom. IQL: Image Quality Level, ROI: region of interest

During the measurements, both direct and indirect radiation dose parameters were recorded. These included the volume CT dose index (CTDI_vol_) and dose-length product (DLP). Additionally, organ-specific radiation doses were calculated by an automated dose monitoring software (Radimetrics Enterprise Platform, Bayer Healthcare, Leverkusen, Germany) applying Monte Carlo simulation techniques. Quantitative image quality assessment was performed by evaluating signal and noise in the brain parenchyma and bone-equivalent regions of the phantoms. For this purpose, signal intensity and standard deviation (as a measure of image noise) were derived from defined regions of interest (ROIs). Signal and noise measurements were obtained using the previously described ROI-based approach [24], with a 5 cm² ROI in the brain-equivalent material and a 1 cm² ROI in the sphenoid bone equivalent (Fig. 1). From these measurements, the SNR was calculated for each IQL. To account for different reconstructed slice thicknesses between the EID-CT (1.0 mm) and the PCCT (1.5 mm) datasets (Table 1), noise values from the EID-CT images were adjusted using the established relationship between noise and slice thickness described by Kanal et al. [25]. According to this approach, image noise scales with the inverse square root of the slice thickness. Therefore, EID-CT noise measured at 1.0 mm was converted to the corresponding noise expected at 1.5 mm slice thickness using the following formula:

$$\begin{array}{c}{Noise}_{EID-CT,\:1.5 mm}={Noise}_{EID-CT,\:1.0 mm}\:\times\:\sqrt{\frac{1.0}{1.5}}\approx\:0.8165\:\times\:\:{Noise}_{EID-CT,\:1.0 mm}\end{array}\:$$

### Statistical analysis

All statistical analyses were performed using SPSS software (version 31.0; IBM Corp., New York, USA). Given the small sample size (*n* < 30) and the resulting limited power for normality testing [26], no assumption of normal distribution was made. Data are therefore reported as median values with corresponding interquartile ranges (IQR). Group comparisons were restricted to identical IQLs, enabling paired analyses via the Wilcoxon signed-rank test. Statistical significance was set at a two-tailed *p* < 0.05. Percent differences between EID-CT and PCCT scans were calculated based on the area under the curve (AUC), determined via trapezoidal integration using Python (version 3.11).

## Results

First, the relationship between radiation dose parameters and IQL was compared between EID-CT and PCCT. Second, image noise and SNR values for brain parenchyma and bone were compared between the two systems.

### Radiation dose metrics

**Fig. 2:**
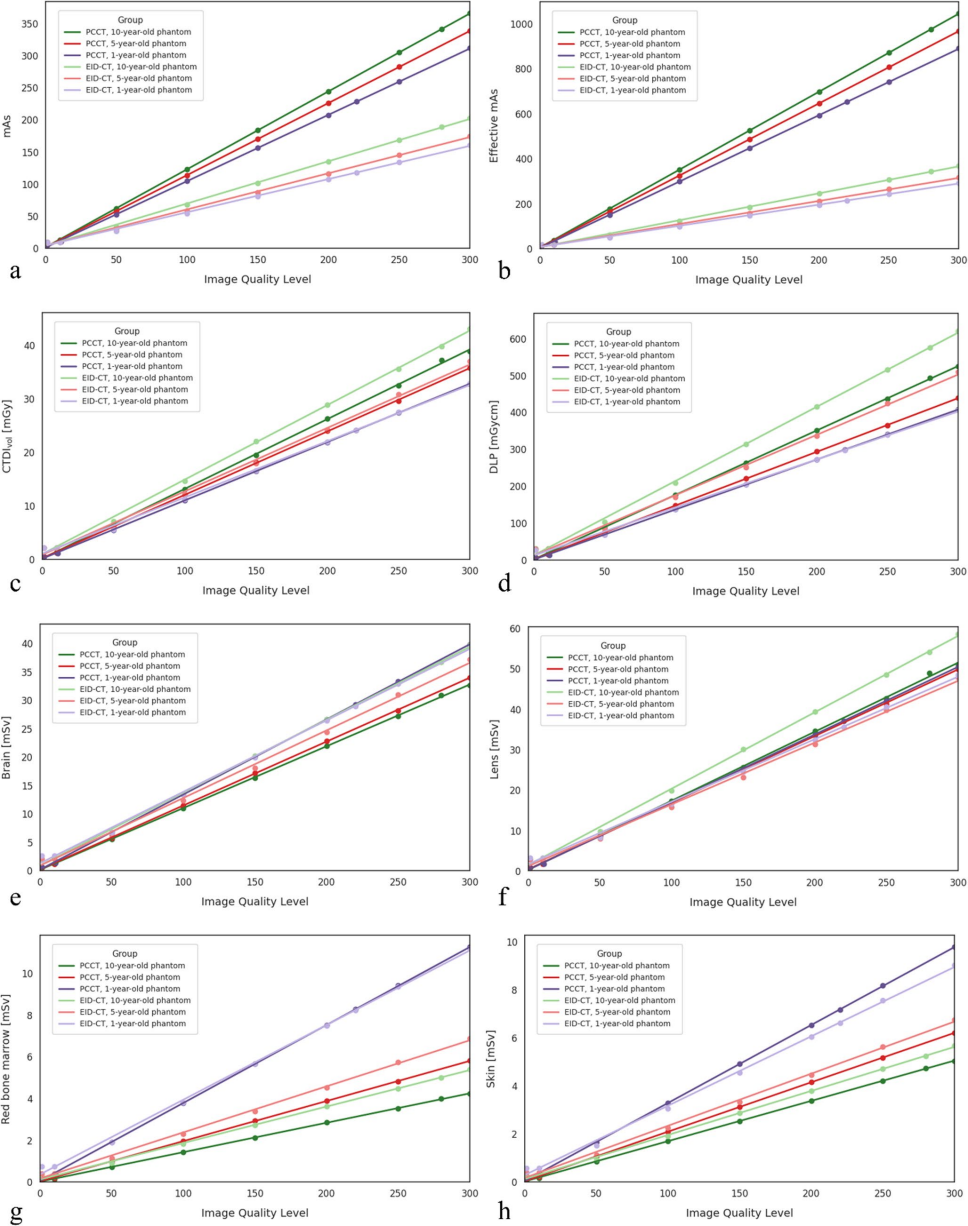
Linear relationship between IQLs and various radiation dose parameters across different phantoms on both EID-CT and PCCT. Pediatric PCCT data have previously been reported in an analysis of age-related differences between adult and pediatric head phantoms [24]. The present analysis addresses a distinct research question by comparing these data with newly acquired matched pediatric EID-CT measurements. **a**: Relationship between IQL and mAs, **b**: Relationship between IQL and effective mAs, **c**: Relationship between IQL and CTDI_vol_. **d**: Relationship between IQL and DLP. **e**: Relationship between IQL and the organ dose for the brain. **f**: Relationship between IQL and the organ dose for the lens. **g**: Relationship between IQL and the organ dose for the red bone marrow. **h**: Relationship between IQL and the organ dose for the skin. CTDI_vol_: Computed Tomography Dose Index volumetric, DLP: Dose Length Product, EID-CT: Energy-Integrating Detector CT, IQL: Image Quality Level, PCCT: Photon-Counting CT

Across all pediatric phantom sizes, PCCT acquisitions required a significantly higher tube current-time product (mAs) compared to EID-CT (*p* = 0.017) but exhibited a lower radiation dose in terms of CTDI_vol_ (Fig. 2c; Table 2). In the 1-year-old phantom, CTDI_vol_ was marginally but significantly lower with PCCT (*p* = 0.011). The relative difference was-49% at IQL 10 (Table 3) and narrowed to 0.45% at IQL 200. Similar trends were observed for the 5-year-old phantom with significantly lower CTDI_vol_ with PCCT (*p* = 0.042, Table 2), while showing a −43% difference at IQL 10 and only 0.8% at IQL 200 (Table 3). In the 10-year-old phantom, differences were more pronounced (*p* = 0.012, Table 2), with 38% lower CTDI_vol_ with PCCT at IQL 10 and still 9% lower CTDI_vol_ at IQL 200 (Table 3).

**Table 2:** Comparison of different dose parameters and SNR between age-specific phantoms scanned on both EID-CT and PCCT

Parameter	Median (IQR) and *p* value) for the 1-year-old phantom			Median (IQR) and *p* value for the 5-year-old phantom			Median (IQR) and *p* value for the 10-year-old phantom		
	EID-CT	PCCT	*p*-value	EID-CT	PCCT	*p* value	EID-CT	PCCT	*p* value
mAs	67.39 (113.45)	130.49 (225.30)	**0.025**	72.86 (123.61)	142.05 (245.34)	**0.025**	84.55 (144.58)	153.49 (264.92)	**0.025**
Effective mAs	122.52 (206.28)	372.83 (643.71)	**0.017**	132.46 (224.74)	405.85 (700.98)	**0.017**	153.74 (262.87)	438.55 (756.93)	**0.017**
CTDI_vol_ [mGy]	13.90 (23.09)	13.75 (23.81)	**0.011**	15.05 (25.97)	15.10 (25.76)	**0.042**	18.35 (30.50)	16.30 (28.28)	**0.012**
DLP [mGy·cm]	171.50 (286.00)	171.00 (296.15)	0.400	210.50 (358.40)	184.50 (317.33)	**0.012**	261.50 (441.03)	219.50 (379.85)	**0.012**
Scan length [mm]	123.31 (2.12)	124.42 (0.31)	**0.002**	137.62 (1.84)	122.43 (0.51)	**< 0.001**	144.00 (0.94)	134.41 (0.86)	**< 0.001**
Brain [mSv]	16.66 (27.72)	16.72 (28.95)	0.889	15.20 (26.12)	14.35 (24.53)	**0.012**	16.84 (28.18)	13.67 (23.68)	**0.012**
Lens [mSv]	20.46 (33.99)	21.12 (36.57)	0.484	19.41 (33.48)	21.02 (35.87)	0.123	24.91 (41.43)	21.37 (37.07)	**0.012**
Red bone marrow [mSv]	4.74 (7.89)	4.72 (8.18)	0.779	2.84 (4.85)	2.45 (4.20)	**0.012**	2.28 (3.84)	1.78 (3.07)	**0.012**
Skin [mSv]	3.82 (6.37)	4.11 (7.11)	0.161	2.80 (4.77)	2.61 (4.50)	**0.012**	2.39 (4.03)	2.11 (3.66)	**0.012**
Effective dose [mSv]	1.24 (2.07)	1.18 (2.05)	**0.012**	0.93 (1.58)	0.79 (1.35)	**0.012**	0.88 (1.48)	0.70 (1.21)	**0.012**
SNR _brain parenchyma_	6.85 (5.37)	7.71 (6.39)	**0.017**	5.62 (4.80)	6.71 (5.80)	**0.025**	5.61 (3.79)	6.70 (4.61)	**0.025**
SNR _bone_	18.82 (14.58)	29.06 (15.21)	**0.012**	20.33 (9.18)	25.73 (10.78)	**0.012**	16.54 (5.95)	22.75 (4.64)	**0.012**
Signal _brain parenchyma_	53.00 (0.75)	54.00 (2.50)	0.943	45.00 (0.75)	46.00 (3.00)	0.914	48.00 (1.75)	50.00 (3.25)	0.666
Noise _brain parenchyma_	7.76 (9.19)	7.00 (7.00)	**0.025**	8.17 (10.21)	7.00 (9.50)	**0.012**	8.57 (8.37)	7.50 (7.00)	0.093
Signal _bone_	705.50 (5.75)	928.50 (10.25)	**0.012**	828.50 (4.25)	1077.50 (10.00)	**0.011**	829.50 (10.75)	1114.50 (12.00)	**0.012**
Noise _bone_	38.38 (35.52)	32.50 (18.25)	**0.012**	41.23 (19.39)	42.00 (15.75)	0.161	50.62 (16.13)	49.00 (8.25)	0.889

Comparison of the same IQLs within the same IQL-range (1–300) for each group (n = 8 per group) with the Wilcoxon-signed rank test. Comparison of scan length by using Mann-Whitney-U test. Pediatric PCCT data have previously been reported [24]; pediatric EID-CT data were newly acquired for the present comparison. EID-CT: Energy-Integrating Detector CT, IQL: Image Quality Level, IQR: Interquartile Range, PCCT: Photon-Counting CT

**Table 3:** CTDI_vol_ for pediatric phantoms (1-year-old, 5-year-old, 10-year-old) at different IQLs

IQL	CTDI_vol_ [mGy]								
	1-year-old phantom, EID-CT	1-year-old phantom, PCCT	% change from EID-CT	5-year-old phantom, EID-CT	5-year-old phantom, PCCT	% Difference	10-year-old phantom, EID-CT	10-year-old phantom, PCCT	% Difference
1	2.18	0.55	−74.77	2.18	0.55	−74.77	2.17	0.55	−74.65
10	**2.18**	**1.11**	**−49.08**	**2.17**	**1.23**	**−43.32**	**2.18**	**1.35**	**−38.07**
50	5.61	5.52	−1.60	6.18	6.08	−1.62	7.15	6.65	−6.99
100	**11.2**	**11**	**−1.79**	**12.2**	**12.1**	**−0.82**	**14.6**	**13.1**	**−10.27**
150	16.6	16.5	−0.60	17.9	18.1	1.12	22.1	19.5	−11.76
200	**22**	**21.9**	**−0.45**	**24.2**	**24**	**−0.83**	**28.9**	**26.3**	**−9.00**
250	27.5	27.4	−0.36	30.8	29.6	−3.90	35.6	32.5	−8.71
300	32.9	32.8	−0.30	37	35.8	−3.24	43.1	38.9	−9.74

% Change calculated as (PCCT – EID-CT)/EID-CT × 100%. EID-CT: Energy-Integrating Detector CT. IQL: Image Quality Level. PCCT: Photon-counting CT

As scan lengths differed significantly between PCCT and EID-CT protocols (Table 2), radiation dose comparisons were restricted to CTDI_vol_. As those differences in DLP were small on average in the 1-year-old phantom (0.3%), they corresponded to non-significant differences in organ doses. Across phantoms, CTDI_vol_ and DLP increased with phantom size, as expected. Notably, the 1-year-old phantom had the highest estimated organ doses for skin and red bone marrow across all phantoms (Fig. 2g-h).

### Image quality

**Fig. 3:**
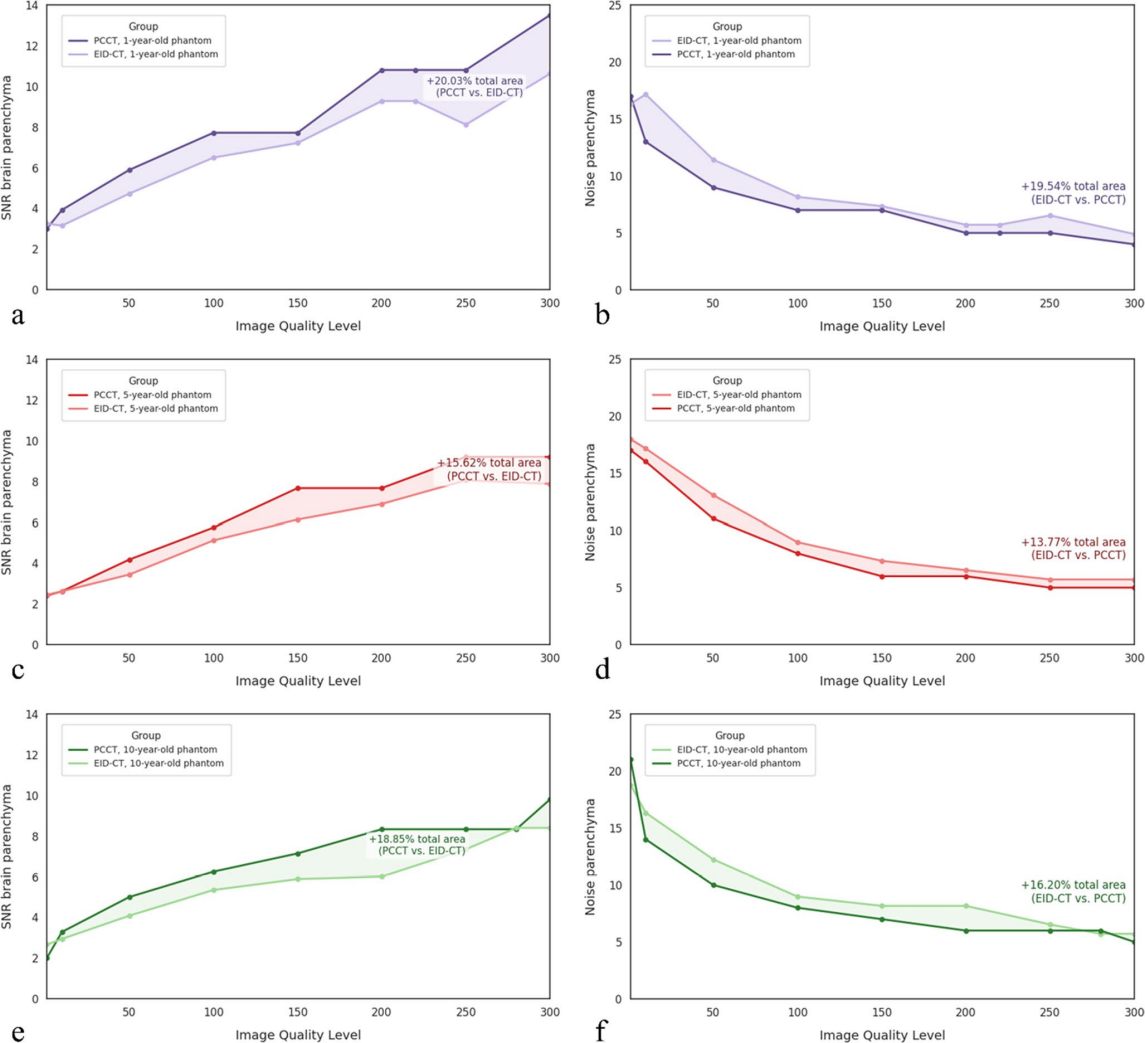
Relationship between IQL and SNR and image noise for brain parenchyma on both EID-CT and PCCT. Pediatric PCCT data have previously been reported [24]; pediatric EID-CT data were newly acquired for the present comparison. **a**: Comparison within the 1-year-old pediatric phantom on both CT scanners for SNR. **b**: Comparison within the 1-year-old pediatric phantom on both CT scanners for image noise. **c**: Comparison within the 5-year-old pediatric phantom on both CT scanners for SNR. **d**: Comparison within the 5-year-old pediatric phantom on both CT scanners for image noise. **e**: Comparison within the 10-year-old pediatric phantom on both CT scanners for SNR. **f**: Comparison within the 10-year-old pediatric phantom on both CT scanners for image noise. EID-CT: Energy-Integrating Detector CT, IQL: Image Quality Level, PCCT: Photon-Counting CT, SNR: signal-to-noise ratio

The comparison of image noise and SNR in brain parenchyma across increasing IQLs revealed four key findings (Fig. 3). First, the relationship between IQL and image noise as well as between IQL and SNR was less linear than that of the radiation dose metrics, with both PCCT and EID-CT showing intermediate plateaus rather than linearly decreasing noise or linearly increasing SNR. Second, within each age-specific phantom, PCCT consistently produced lower image noise and higher SNR for brain parenchyma than the corresponding EID-CT acquisition from IQL 10 onward (Tables 4, 5 and 6). These differences were statistically significant for SNR of brain parenchyma for all pediatric phantoms (Table 2; *p* = 0.017 for the 1-year-old phantom, *p* = 0.025 for the 5- and 10-year-old phantom). Regarding image noise, significant differences between EID-CT and PCCT were observed only for the 1- and 5-year-old phantoms (Table 2; *p* = 0.025 for the 1-year-old phantom, *p* = 0.012 for the 5-year-old phantom). Third, younger age-specific phantoms exhibited higher SNR values and lower image noise than older age-specific phantoms, regardless of scanner type (*p* < 0.05). Fourth, the largest relative difference between PCCT and EID-CT was observed in the 1-year-old phantom (+ 20.03% AUC over the full IQL-range of SNR and − 19.54% for image noise), followed by the 10-year-old phantom (+ 18.85% for SNR and − 16.20% for image noise) and 5-year-old phantom (+ 15.62% for SNR and − 13.77% for image noise) (Fig. 3).

**Table 4:** SNR and image noise for brain parenchyma and bone for the 1-year-old pediatric phantoms at different IQLs after correction for different reconstructed slice thicknesses

IQL	SNR_brain parenchyma_			SNR_bone_		
	1-year-old phantom, EID-CT	1-year-old phantom, PCCT	% Difference	1-year-old phantom, EID-CT	1-year-old phantom, PCCT	% Difference
1	3.25	3.00	−7.57	10.39	21.09	103.03
10	**3.15**	**3.92**	**24.57**	**10.64**	**22.33**	**109.81**
50	4.72	5.89	24.66	12.89	21.27	65.07
100	**6.49**	**7.71**	**18.84**	**16.08**	**25.00**	**55.47**
150	7.21	7.71	6.96	21.56	33.11	53.59
200	**9.27**	**10.80**	**16.47**	**21.62**	**35.96**	**66.36**
250	8.11	10.80	33.10	27.17	37.16	36.75
300	10.61	13.50	27.19	27.21	37.00	35.97
	Noise _brain parenchyma_			Noise _bone_		
IQL	1-year-old phantom, EID-CT	1-year-old phantom, PCCT	% difference	1-year-old phantom, EID-CT	1-year-old phantom, PCCT	% difference
1	16.33	17.00	4.10	67.77	44.00	−35.07
10	**17.15**	**13.00**	**−24.18**	**66.14**	**42.00**	**−36.49**
50	11.43	9.00	−21.27	54.71	44.00	−19.57
100	**8.16**	**7.00**	**−14.27**	**44.09**	**37.00**	**−16.08**
150	7.35	7.00	−4.74	32.66	28.00	−14.27
200	**5.72**	**5.00**	**−12.52**	**32.66**	**26.00**	**−20.39**
250	6.53	5.00	−23.45	26.13	25.00	−4.32
300	4.90	4.00	−18.35	26.13	25.00	−4.32

% Change calculated as (PCCT – EID-CT)/EID-CT × 100%. EID-CT: Energy-Integrating Detector CT. IQL: Image Quality Level. PCCT: Photon-counting CT

**Table 5:** SNR and image noise for brain parenchyma and bone for the 5-year-old pediatric phantoms at different IQLs after correction for different reconstructed slice thicknesses

IQL	SNR_brain parenchyma_			SNR_bone_		
	5-year-old phantom, EID-CT	5-year-old phantom, PCCT	% Difference	5-year-old phantom, EID-CT	5-year-old phantom, PCCT	% Difference
1	2.45	2.41	−1.54	15.81	21.64	36.90
10	**2.62**	**2.63**	**0.02**	**15.58**	**25.21**	**61.78**
50	3.44	4.18	21.40	12.37	20.65	67.01
100	**5.12**	**5.75**	**12.27**	**18.17**	**24.91**	**37.04**
150	6.12	7.67	25.20	22.48	26.24	16.74
200	**6.89**	**7.67**	**11.29**	**23.13**	**31.79**	**37.45**
250	8.05	9.20	14.31	25.38	33.72	32.84
300	7.87	9.20	16.85	27.44	34.55	25.90
	Noise _brain parenchyma_			Noise _bone_		
IQL	5-year-old phantom, EID-CT	5-year-old phantom, PCCT	% difference	5-year-old phantom, EID-CT	5-year-old phantom, PCCT	% difference
1	17.96	17.00	−5.36	52.26	50.00	−4.32
10	**17.15**	**16.00**	**−6.69**	**53.07**	**43.00**	**−18.98**
50	13.06	11.00	−15.80	66.95	52.00	−22.33
100	**8.98**	**8.00**	**−10.93**	**45.72**	**43.00**	**−5.96**
150	7.35	6.00	−18.35	36.74	41.00	11.59
200	**6.53**	**6.00**	**−8.14**	**35.93**	**34.00**	**−5.36**
250	5.72	5.00	−12.52	32.66	32.00	−2.02
300	5.72	5.00	−12.52	30.21	31.00	2.61

% Change calculated as (PCCT – EID-CT)/EID-CT × 100%. EID-CT: Energy-Integrating Detector CT. IQL: Image Quality Level. PCCT: Photon-counting CT

**Table 6:** SNR and image noise for brain parenchyma and bone for the 10-year-old pediatric phantoms at different IQLs after correction for different reconstructed slice thicknesses

IQL	SNR_brain parenchyma_			SNR_bone_		
	10-year-old phantom, EID-CT	10-year-old phantom, PCCT	% Difference	10-year-old phantom, EID-CT	10-year-old phantom, PCCT	% Difference
1	2.66	2.00	−24.88	15.28	16.98	11.14
10	**2.94**	**3.29**	**11.78**	**14.99**	**22.02**	**46.95**
50	4.08	5.00	22.47	12.63	22.32	76.72
100	**5.34**	**6.25**	**16.95**	**15.26**	**22.30**	**46.10**
150	5.88	7.14	21.50	17.81	23.17	30.06
200	**6.00**	**8.33**	**38.86**	**19.93**	**25.32**	**27.02**
250	7.35	8.33	13.40	21.37	27.24	27.50
300	8.40	9.80	16.69	23.44	27.20	16.01
	Noise _brain parenchyma_			Noise _bone_		
IQL	10-year-old phantom, EID-CT	10-year-old phantom, PCCT	% difference	10-year-old phantom, EID-CT	10-year-old phantom, PCCT	% difference
1	18.78	21.00	11.82	54.71	64.00	16.99
10	**16.33**	**14.00**	**−14.27**	**55.52**	**50.00**	**−9.95**
50	12.25	10.00	−18.35	65.32	50.00	−23.45
100	**8.98**	**8.00**	**−10.93**	**54.71**	**50.00**	**−8.60**
150	8.16	7.00	−14.27	46.54	48.00	3.14
200	**8.16**	**6.00**	**−26.52**	**41.64**	**44.00**	**5.66**
250	6.53	6.00	−8.14	38.38	41.00	6.84
300	5.72	5.00	−12.52	35.11	41.00	16.78

% Change calculated as (PCCT – EID-CT)/EID-CT × 100%. EID-CT: Energy-Integrating Detector CT. IQL: Image Quality Level. PCCT: Photon-counting CT

**Fig. 4:**
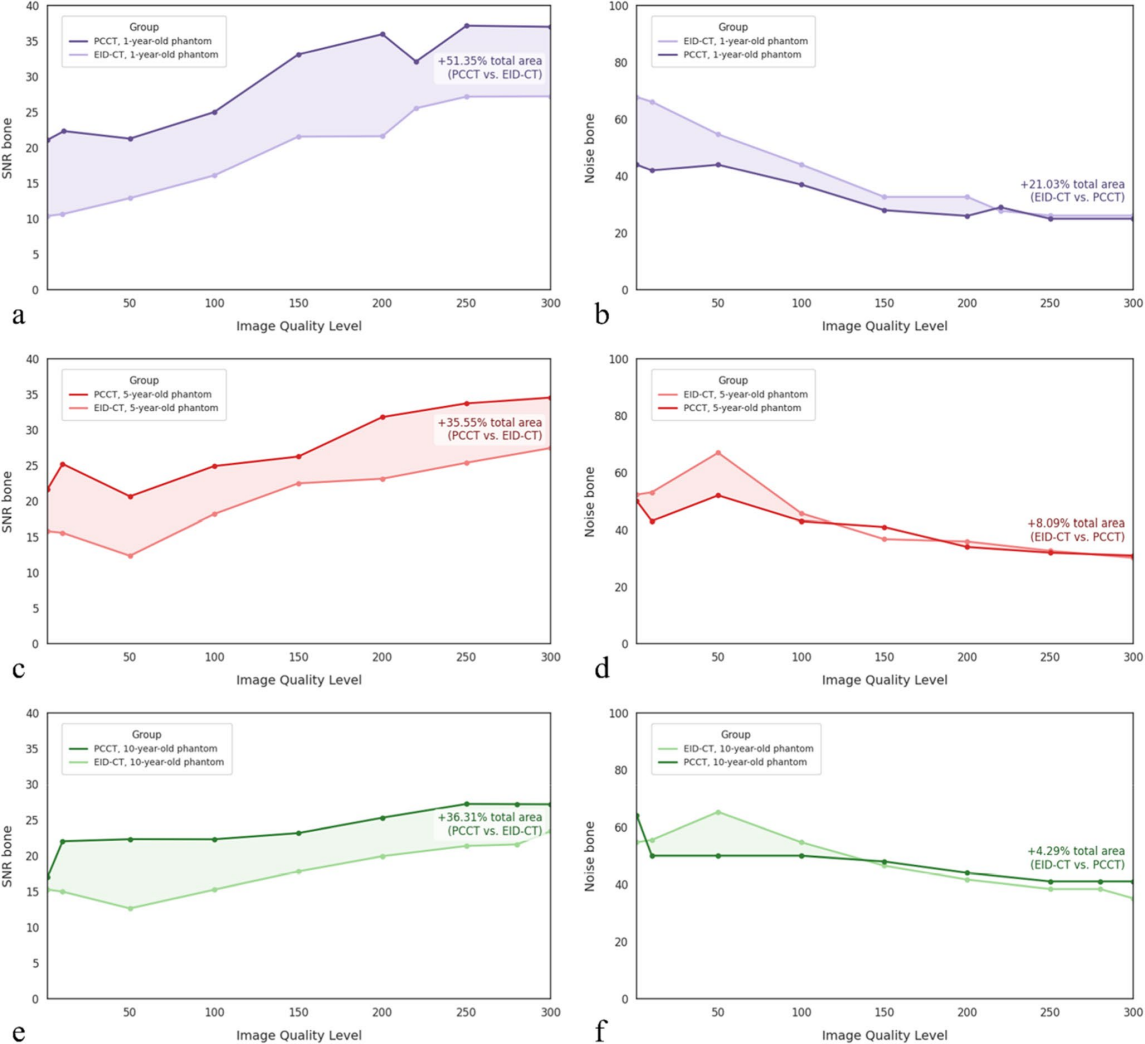
Comparison between IQL and SNR and image noise for bone on both EID-CT and PCCT. Pediatric PCCT data have previously been reported [24]; pediatric EID-CT data were newly acquired for the present comparison. **a**: Comparison within the 1-year-old pediatric phantom on both CT scanners for SNR. **b**: Comparison within the 1-year-old pediatric phantom on both CT scanners for image noise. **c**: Comparison within the 5-year-old pediatric phantom on both CT scanners for SNR. **d**: Comparison within the 5-year-old pediatric phantom on both CT scanners for image noise. **e**: Comparison within the 10-year-old pediatric phantom on both CT scanners for SNR. **f**: Comparison within the 10-year-old pediatric phantom on both CT scanners for image noise. EID-CT: Energy-Integrating Detector CT, IQL: Image Quality Level, PCCT: Photon-Counting CT, SNR: signal-to-noise ratio

The analysis of SNR in bone across increasing IQLs revealed several consistent patterns (Fig. 4). First, bone SNR values were higher than those for brain parenchyma in all measurements. Second, the rate of SNR increase with rising IQLs was less pronounced for bone than for parenchyma. Third, younger age phantoms generally exhibited higher bone SNR values than older phantoms, regardless of scanner type. However, differences in image noise between the 5- and 10-year-old phantoms were less consistent, with PCCT showing lower noise only up to approximately IQL 100 (Tables 4, 5 and 6). Over the full IQL-range, significant noise differences between EID-CT and PCCT were observed only for the 1-year-old phantom (Table 2; *p* = 0.012). Fourth, differences between PCCT and EID-CT were more pronounced for bone than for brain parenchyma, with the largest relative AUC gain observed in the 1-year-old phantom (+ 51.35%), followed by the 10-year-old (+ 36.31%) and 5-year-old (+ 35.55%). These differences were statistically significant for all pediatric phantoms (Table 2; *p* = 0.012).

## Discussion

This study investigated the relationship between IQL, radiation dose, image noise, and SNR in pediatric head CT imaging using phantom models scanned with both EID-CT and PCCT systems. Standard clinical protocols for each system were applied, allowing a direct comparison under realistic clinical conditions.

### Radiation dose

A strictly linear relationship between IQL and radiation dose metrics was observed for both detector technologies, in agreement with prior studies [21]. This supports the robustness of IQL as a reproducible, system-independent measure for dose modulation in PCCT imaging. In contrast, the relationship between IQL and both SNR and image noise was less linear, showing intermediate plateaus. Earlier studies have reported a quadratic relationship in the literature [22]. The intermediate plateau regions may be attributed to the nonlinear noise reduction achieved with iterative reconstruction algorithms, resulting in progressively smaller SNR improvements at higher dose levels [27–29]. Similar trends have also been observed in adult head phantoms [24]. From a clinical perspective, this implies that in the upper IQL-range, further increases in radiation exposure result in progressively smaller gains in SNR or markedly slower reduction in image noise.

Despite PCCT acquisitions requiring significantly higher tube current-time products (mAs) than EID-CT, radiation dose values expressed as CTDI_vol_ and DLP were lower for PCCT across all phantom sizes. This can be explained by the different tube voltages used (70 kVp for PCCT vs. 100 kVp for EID-CT) as radiation exposure increases approximately with the square of the tube voltage [28]. Consequently, the lower kVp in PCCT necessitated higher mAs to maintain target image quality. Directly comparing both systems at matched kVp (e.g., 70 kVp) could provide further insights into pure detector-related dose efficiency.

The relative dose advantage of PCCT increased with phantom size: while differences in CTDI_vol_ in the high IQL-range (IQL 200–300) were negligible in the 1-year-old phantom (0.3%), they reached 8–10% in the 10-year-old phantom. This suggests that PCCT’s dose efficiency may be more pronounced in larger pediatric patients, potentially due to improved photon utilization at lower kVp.

Differences in DLP between the two systems were partly attributable to significant variations in scan length limiting the comparison especially for the 5- and 10-year-old phantoms. Organ doses also showed a linear relationship with increasing IQL. Notably, the red bone marrow dose in the 1-year-old phantom was substantially higher than in older age groups. This reflects the different distribution of hematopoietic bone marrow: in newborns, approximately 30% of bone marrow is located in the skull, whereas by early adulthood this proportion decreases to about 8% [30, 31]. The ATOM phantom models accurately account for this distribution, providing a realistic simulation of bone marrow [23]. This underscores the critical importance of radiation dose considerations in very young children as it is known that small children have higher conversion factors from DLP to effective dose compared to older children or adults due to their smaller body size and greater radiation sensitivity [32].

### Image noise and SNR

Across all pediatric phantom sizes and IQL settings, PCCT consistently produced higher SNR than EID-CT, even after mathematically accounting for differences in slice thickness, with statistically significant differences in SNR for both brain parenchyma and bone. Importantly, potential confounding factors such as reconstruction kernel and iterative reconstruction level were kept constant, suggesting that these improvements are most likely attributable to intrinsic advantages of PCCT technology, including reduced electronic noise, improved photon counting efficiency, and the elimination of electronic integration noise inherent to EID systems [5].

In brain parenchyma, the SNR advantage of PCCT was primarily driven by reduced image noise, as signal intensity did not differ substantially between the two systems. Accordingly, the shape of the SNR curves closely mirrored the noise curves: larger differences in noise translated directly into larger differences in the resulting SNR. In contrast, for bone, differences in image noise were only evident at lower IQLs (approximately up to IQL 100). Nevertheless, SNR remained consistently higher for PCCT across the entire IQL-range. This reflects the fact that differences for bone SNR were influenced not only by noise but also by scanner- and kVp-dependent differences in signal intensity as the two scanners operated at different tube voltages (70 kVp for PCCT vs. 100 kVp for EID-CT). In contrast to brain parenchyma, bone attenuation is strongly influenced by photon energy due to its higher effective atomic number in calcium-rich tissue and is approximately proportional to Z³/E³ (with Z being the atomic number and E the photon energy) [33]. This effect plays a smaller role in soft tissues such as brain parenchyma, accounting for the absence of significant signal differences in that compartment. For this reason, noise values alone are insufficient for a meaningful comparison. Using SNR for bone provides a more appropriate metric, as it reflects both signal changes arising from kVp-dependent attenuation and the accompanying noise.

The relative SNR advantage of PCCT over EID-CT was more pronounced for bone than for brain parenchyma, in line with prior studies demonstrating the strength of PCCT for high-contrast osseous imaging [9–13]. Further, the 1-year-old phantom showed lower signal in bone compared to the older pediatric models. The lower bone signal observed in the younger phantom is related to age-dependent differences in phantom composition. Reduced mineralization of pediatric bone results in lower radiodensity and may therefore lead to lower CT signal values [23]. These findings also indicate that quantitative image quality metrics should be interpreted separately for different tissue types.

In terms of practical recommendations based on these initial data, we provide only tentative, SNR‑based guidance for both scanners: For brain parenchyma, intermediate SNR plateaus begin at different IQLs depending on phantom age: around IQL 100 for the 1-year-old phantom, IQL 150 for the 5-year-old phantom, and IQL 200 for the 10-year-old phantom. Beyond these points, SNR continues to increase slightly, but the gain is no longer proportional to the roughly linear increase in radiation dose. For bone structures, the SNR gain curve is much flatter. In the 10-year-old phantom, SNR shows minimal improvement beyond IQL ≈ 20, whereas in the 1-year-old phantom, a rapid doubling of SNR can still be observed. Therefore, higher IQLs should be evaluated using additional criteria such as CNR, lesion detectability, or subjective image quality to determine whether further increases meaningfully improve diagnostic image quality.

### Limitations

This study is not without limitations. First, the analysis is solely based on phantom measurements, which limits the direct transferability of results to clinical settings. In particular, the considerable variation in head size within the age range of 0-5 years could not be adequately captured in this phantom study, as only two ATOM phantoms for this age group (a 1-year-old and a 5-year-old phantom) were available for this study [34, 35]. However, while some studies have already compared image quality between EID-CT and PCCT in adult patients [17, 36], repeated systematic measurements in children are ethically not feasible. Therefore, pediatric phantoms represent the only viable option for a systematic assessment of differences between EID-CT and PCCT across a range of IQLs in this population. Second, the applicability of contrast-to-noise ratio (CNR) as an image quality metric was limited because the phantoms do not provide intrinsic contrast differences between tissues. While SNR provides valuable objective information, CNR is more directly linked to diagnostic performance. Phantoms are useful tools for repeated radiation dose measurements at different settings while still allowing objective image quality measurements; however, more dedicated methods, such as the use of a modulation transfer function (MTF) to measure spatial resolution, were not possible with this study approach. Future studies should evaluate the diagnostic relevance of these findings using complementary approaches, such as subjective reader assessments and lesion detectability analyses. In addition, no repeated scans were performed at the same IQL settings. Given the high degree of standardization in phantom composition, repeated measurements would be expected to produce nearly identical results. The consistency of the observed trends across all phantom sizes and IQLs further supports the robustness of the findings despite the absence of repeated acquisitions. Third, although the majority of reconstruction parameters for EID-CT and PCCT were kept constant, the slice thickness differed between the two systems. As thinner slices lead to higher image noise, an additional mathematical conversion was applied to account for this difference. While this correction is only an approximation and does not fully eliminate all slice-thickness effects, PCCT still demonstrated higher SNR and image quality compared to EID-CT. Therefore, general conclusions regarding image quality can still be drawn, although further studies are warranted to confirm these findings under fully standardized conditions. Finally, the comparison was limited to a single PCCT and a single EID-CT system. This reflects current market availability, as the Siemens PCCT platform is presently the only commercially available photon-counting CT scanner. The chosen EID-CT comparator (Somatom X.ceed) represents a high-end dual-energy system and therefore serves as a strong reference standard; however, the results may not be directly generalizable to other EID-CT models or vendors.

## Conclusion

Our results suggest that while PCCT can reduce radiation dose compared to EID-CT, its advantage in pediatric head imaging might also lie in reduced image noise and higher SNR, particularly for osseous structures, without compromising radiation dose efficiency. Given the nonlinear relationship between radiation dose and SNR, careful protocol optimization is essential to avoid unnecessary dose increases that yield only minimal image quality benefits. For pediatric neuroimaging, PCCT offers a promising balance between dose reduction and diagnostic performance, especially in age groups with heightened radiation sensitivity.

## Supplementary Information

Below is the link to the electronic supplementary material.

Supplementary File 1 (DOCX 637 KB)

## Data Availability

The datasets used and analyzed during the current study are available from the corresponding author upon reasonable request.
