# Supplementary material for: Radiation dose and signal-to-noise ratio in pediatric head CT: a phantom study comparing photon-counting and energy-integrating detectors
Source: Neuroradiology. 2026 Jun 20;68(7):1811–23. doi: 10.1007/s00234-026-04075-9 (PMC13407449; doi:10.1007/s00234-026-04075-9)
Supplement: Supplementary file 1 — (DOCX 637 KB) [file 234_2026_4075_MOESM1_ESM.docx]

**Supplementary material**


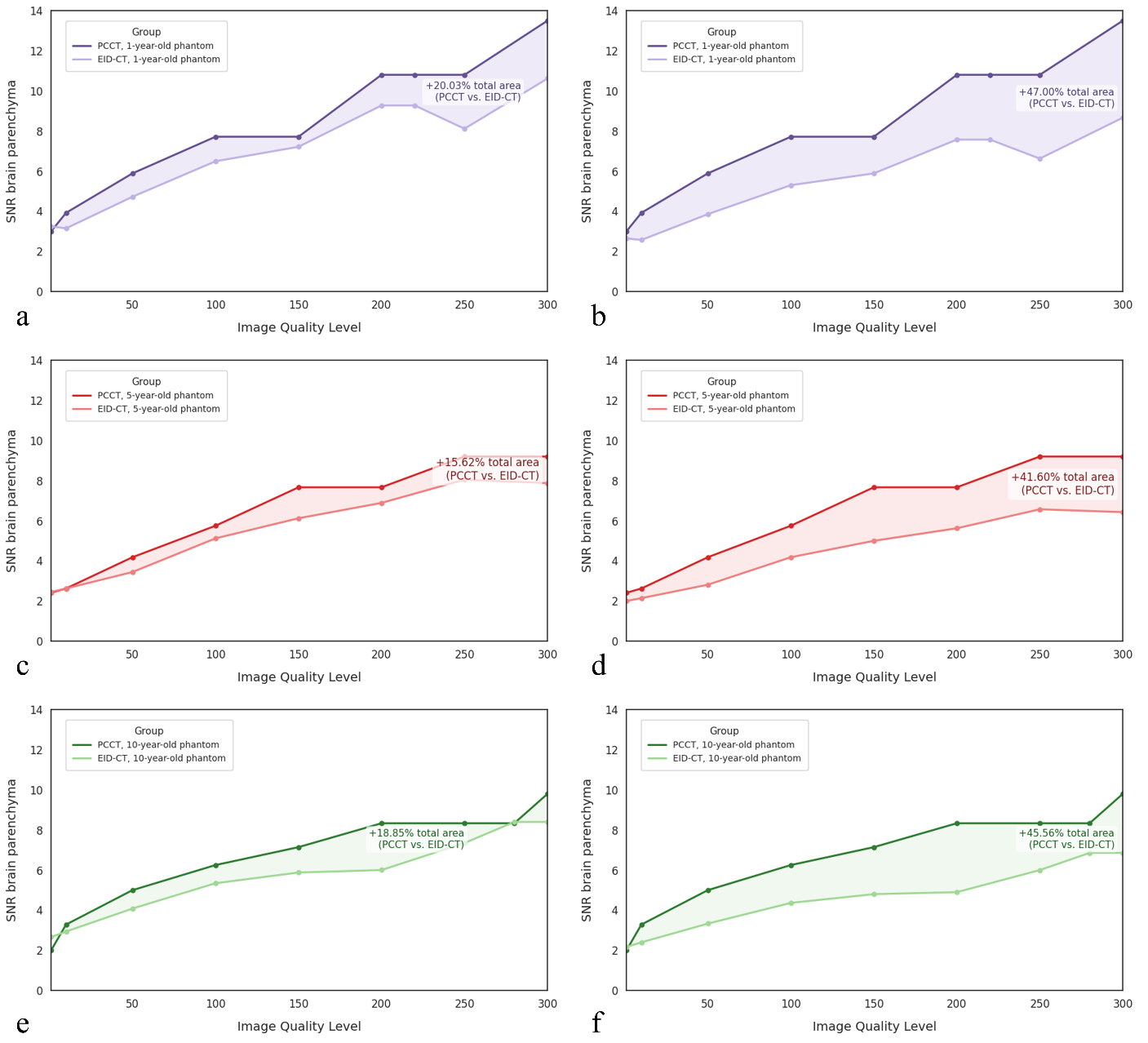


**Figure I**: Relationship between IQL and SNR for brain parenchyma on both EID-CT and PCCT for data before and after correction for different reconstructed slice thickness. Pediatric PCCT data have previously been reported [1]; pediatric EID-CT data were newly acquired for the present comparison. a: Comparison within the 1-year-old pediatric phantom on both CT scanners for corrected slice thickness. b: Comparison within the 1-year-old pediatric phantom on both CT scanners for uncorrected slice thickness. c: Comparison within the 5-year-old pediatric phantom on both CT scanners for corrected slice thickness. d: Comparison within the 5-year-old pediatric phantom on both CT scanners for uncorrected slice thickness. e: Comparison within the 10-year-old pediatric phantom on both CT scanners for corrected slice thickness. f: Comparison within the 10-year-old pediatric phantom on both CT scanners for uncorrected slice thickness. EID-CT: Energy-Integrating Detector CT, IQL: Image Quality Level, PCCT: Photon-Counting CT, SNR: signal-to-noise ratio.


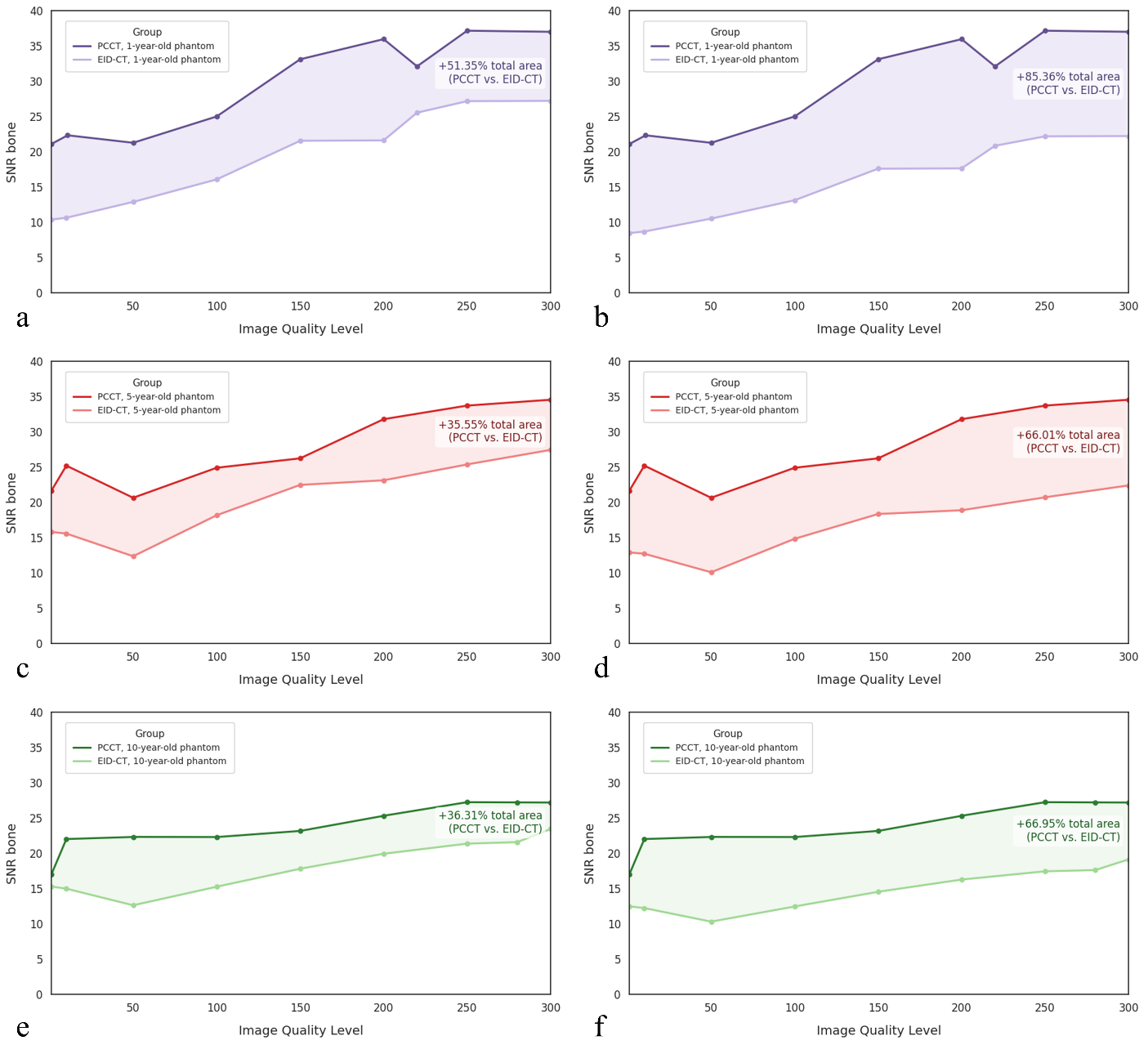


**Figure II**: Relationship between IQL and SNR for bone on both EID-CT and PCCT for data before and after correction for different reconstructed slice thickness. Pediatric PCCT data have previously been reported [1]; pediatric EID-CT data were newly acquired for the present comparison. a: Comparison within the 1-year-old pediatric phantom on both CT scanners for corrected slice thickness. b: Comparison within the 1-year-old pediatric phantom on both CT scanners for uncorrected slice thickness. c: Comparison within the 5-year-old pediatric phantom on both CT scanners for corrected slice thickness. d: Comparison within the 5-year-old pediatric phantom on both CT scanners for uncorrected slice thickness. e: Comparison within the 10-year-old pediatric phantom on both CT scanners for corrected slice thickness. f: Comparison within the 10-year-old pediatric phantom on both CT scanners for uncorrected slice thickness. EID-CT: Energy-Integrating Detector CT, IQL: Image Quality Level, PCCT: Photon-Counting CT, SNR: signal-to-noise ratio.

**Reference**

LV Klüner, MK Opitz, H Peuster et al (2025) Investigating Radiation Dose and Signal-To-Noise-Ratio in Pediatric and Adult Head Photon-Counting CT: A Phantom-Based Study. Rofo. doi: 10.1055/a-2740-4588
